# Perforation of the Meckel’s Diverticulum Following Blunt Trauma to the Abdomen

**DOI:** 10.7759/cureus.12868

**Published:** 2021-01-22

**Authors:** Sandeep Bhattarai, Oseen Shaikh, Muhamed Tajudeen, Uday Kumbhar, Gopal Balasubramanian

**Affiliations:** 1 Surgery, Jawaharlal Institute of Postgraduate Medical Education and Research, Puducherry, IND

**Keywords:** perforation, blunt trauma abdomen, meckel’s diverticulum

## Abstract

Meckel’s diverticulum (MD) is the most common congenital anomaly of the gastrointestinal tract. Most of the patients are asymptomatic and very few develop symptoms. Hemorrhage, obstruction, perforation, and inflammation are the complications that can occur in an MD. Even though hollow viscus perforation is common, perforation of the MD following blunt abdominal trauma is rare. We report a case of perforation of the MD in a 60-year-old man following a blunt abdominal trauma due to a fall from a bike, which was diagnosed promptly and managed successfully by timely operative intervention.

## Introduction

Meckel’s diverticulum (MD) develops due to incomplete obliteration of the omphalomesenteric duct resulting in the formation of a diverticulum in the distal ileum. MD is usually asymptomatic. The lifetime reported complication rate is about 4%, which can be in the form of lower gastrointestinal bleed, intestinal obstruction, perforation, diverticulitis, and rarely malignancy [1]. Bleeding from the MD is the most common presentation in children, whereas intestinal obstruction is common in adults. Perforation of the MD is uncommon and mostly occurs due to the presence of ectopic gastric mucosa. Perforation of the MD following blunt abdominal trauma is very rare. We present a rare case of perforated MD following blunt abdominal trauma.

## Case presentation

**Figure 1:**
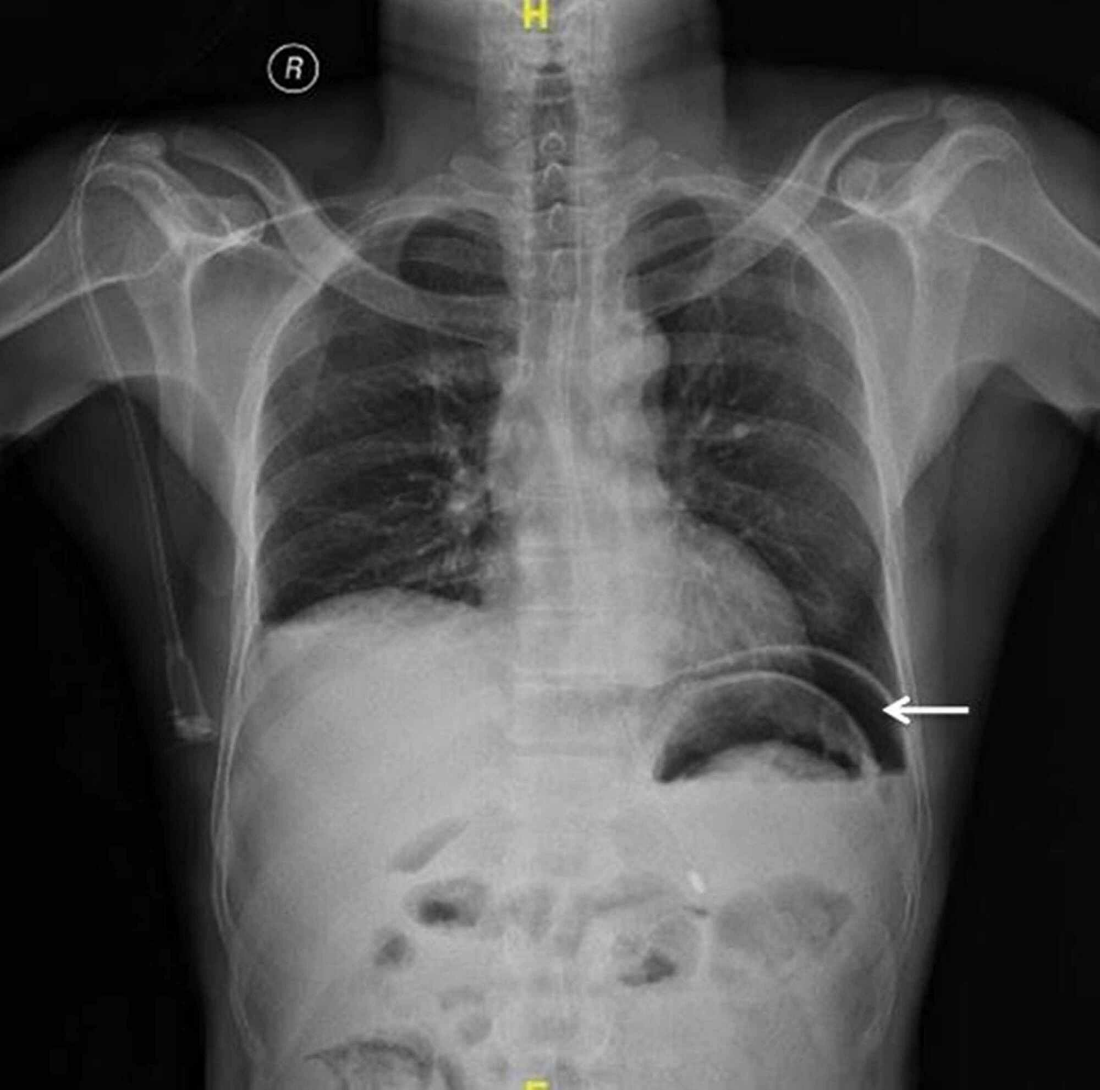
X-ray of the chest and abdomen showing gas under the diaphragm displacing fundus of the stomach.

A 60-year-old male had fallen from the bike while driving and sustained blunt trauma to the abdomen from the bike’s handle. The patient did not sustain injury elsewhere apart from mild trauma to the right elbow. Following trauma, he developed severe abdominal pain and consulted a local private hospital, from where he was referred to us. The patient was referred to our hospital within six hours following trauma. The patient was fully conscious and orientated. His pulse rate was 96 beats/minute and blood pressure was 120/60 mmHg. There were restricted abdominal movements on examination, and there was diffuse tenderness all over the abdomen along with board-like generalized rigidity. There was obliteration of liver dullness. An erect abdominal X-ray revealed gas under the diaphragm along the left hemidiaphragm, displacing the fundus of the stomach downwards (Figure 1).

A focused assessment with sonography for trauma scan was done showing mild free fluid in the abdomen, mainly in the perihepatic region and hepatorenal pouch, without solid organ injury. Diagnostic peritoneal aspiration was done revealing hemorrhagic bilious aspirate. With a diagnosis of perforated hollow viscus with peritonitis, he was taken up for emergency laparotomy.

**Figure 2:**
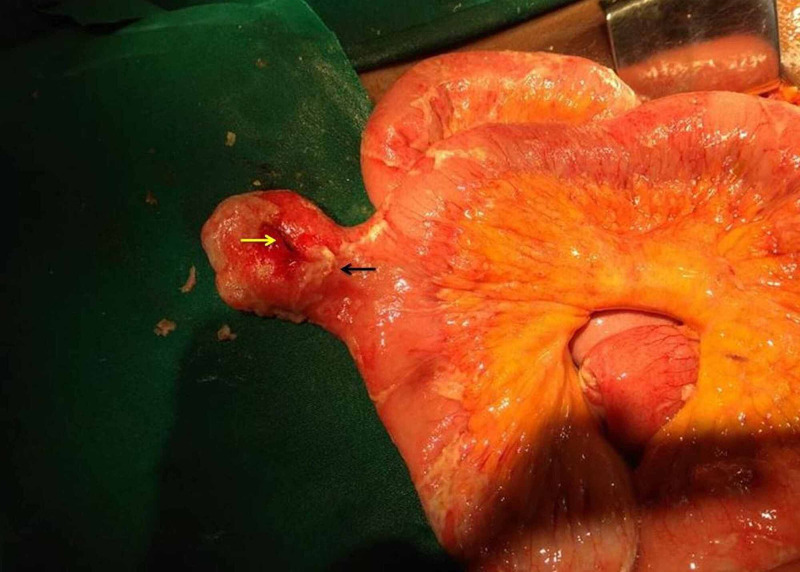
Intraoperative image showing MD along the antimesenteric border (black arrow) with perforation (yellow arrow). MD, Meckel’s diverticulum

A midline incision was taken to open the abdomen. There was bilious free fluid in the abdomen. On thorough bowel examination, MD was found at the ileum’s antimesenteric border of ileum about 60 cm proximal to the ileocecal junction. There was a perforation, measuring 1 cm × 1 cm, approximately 1 cm proximal to the tip of the MD (Figure 2).

There was no evidence of any other perforation or solid organ injury. A wedge resection of the diverticulum followed by primary closure of the ileum was done. The patient improved postoperatively without any complications.

## Discussion

MD is the most common congenital anomaly of the small intestine and follows an orderly occurrence rule of two. MD occurs in 2% of the population, is 60 cm from the ileocecal valve, two inches in length, and contains two types of heterotopic mucosal tissue (gastric and pancreatic, occasionally colonic). The reported lifetime complication rate is 4%.

MD is a true diverticulum containing all three layers of the bowel wall. Failure to obliterate the vitellointestinal duct leads to various developmental anomalies, with MD being the most common [2].

MD is usually asymptomatic and symptoms occur in 2% of the cases [1]. Patients develop symptoms secondary to MD complications such as intestinal obstruction, diverticulitis, intussusception, perforation, or gastrointestinal bleeding. Other complications include volvulus, neoplasm, or may present as litter’s hernia. Intestinal obstruction is the most common presentation in adults, whereas gastrointestinal bleeding is the most common in children [3,4].

Factors associated with increased risk of complications include male sex, age below 50 years, presence of heterotopic mucosa within the diverticulum, length of diverticulum greater than 2 cm, or a diverticular height-to-diameter ratio of greater than two.

Perforation of the MD is rare and found to be in only 7% of the patients [4]. It is mostly due to the presence of ectopic gastric or pancreatic tissue [4]. However, only 60% of the patients with MD are known to have ectopic tissue. A study done by Stein et al. reported ectopic gastric mucosa in 16% of all patients with MD [5].

Perforation of the MD as a consequence of the abdominal trauma is uncommon. There is a literature report where perforation of the MD occurred following penetrating trauma to the abdomen. MD perforation following blunt trauma to the abdomen is very rare. Small bowel injury in a blunt trauma of the abdomen is common. The mechanism of the injury may be either pressure rupture, crushing, or shear force. Pressure rupture occurs when a loop of the bowel closes with the trapped lumen and the squeeze increases internal pressure, causing the bowel wall to give away at a weak point. Crushing injury can occur, and then the direct blow crushes a segment to the relatively fixed and rigid structure such as vertebra. Shear force occurs in the setting of sudden deceleration or acceleration injury, as seen in a fall from height or a road traffic accident, where a sudden halt causes the bowel to keep moving forward where the fixed points like duodenojejunal flexure and ileocaecal junction bear the pull and give away. Seat belt during a deceleration injury adds to the risk of bowel perforation. It is rare for an MD to perforate due to blunt trauma because any ligament or mesentery does not bind it [4].

Thus, acceleration and deceleration injury is not expected. However, it has been proposed that blunt abdominal trauma may cause perforation of the MD, especially if any residual mesodiverticular band is present, which may rupture and tear the diverticulum due to forced movement of abdominal contents [6]. Once there is a perforation, the patient may present with signs of peritonitis. Our patient presented with signs of peritonitis after blunt trauma to the abdomen. Intraoperatively, we noted a perforation in the MD. However, there was no evidence of any band attachment to the MD. We propose that the perforation of the MD following blunt abdominal trauma was due to increased abdominal pressure.

The preoperative diagnosis of MD is difficult [1]. Ultrasound abdomen and computed tomography of the abdomen may help in diagnosis, but none are conclusive. Scintigraphy is helpful when there is bleeding from the MD. However, it is useful only when the ectopic gastric mucosa lines the MD. The perforated MD cannot be diagnosed preoperatively, and most of the patients with evidence of perforation are diagnosed during surgery. In our case, the preoperative diagnosis was hollow viscus perforation, whereas the site of perforation to be MD was diagnosed intraoperatively.

Histopathologically, it is a true diverticulum containing all three layers of the bowel wall (mucosa, submucosa, and muscularis propria). MD may also contain heterotopic rests of gastric or pancreatic tissue, which are likely to increase the chances of complications. All bowel wall layers were present in our patient, but there was no evidence of any heterotopic mucosa. However, there is a possibility that we might have missed the ectopic tissue, which is frequently found on the opposite wall of the MD, as we did a wedge resection of MD.

Surgical management options of symptomatic diverticula include a diverticulectomy or segmental bowel resection with primary anastomosis. A diverticulectomy can be done, provided that the ileal lumen is not compromised. Otherwise, segmental bowel resection is a good option. In our patient, we performed diverticulectomy, and the defect closed in two layers. Factors responsible for our patient’s successful management were early presentation with minimal intraperitoneal contamination, absence of sepsis, and timely surgical intervention.

## Conclusions

MD is mostly asymptomatic, and only a few patients present with symptoms. Perforation of the MD is an uncommon phenomenon that occurs mostly in patients with ectopic gastric tissue. Perforation of the MD following blunt trauma abdomen is extremely rare, and very few cases have been reported in the literature. Preoperative diagnosis of the perforated MD is difficult and is usually diagnosed intraoperatively. Treatment is mainly surgical with diverticulectomy or segmental resection of the bowel.
